# Development and Validation of a Prognostic Model to Predict the Prognosis of Patients With Retroperitoneal Liposarcoma: A Large International Population-Based Cohort Study

**DOI:** 10.3389/fonc.2022.857827

**Published:** 2022-06-02

**Authors:** Yiding Li, Guiling Wu, Yujie Zhang, Wanli Yang, Xiaoqian Wang, Lili Duan, Liaoran Niu, Junfeng Chen, Wei Zhou, Jinqiang Liu, Helun Zhong, Daiming Fan, Liu Hong

**Affiliations:** ^1^State Key Laboratory of Cancer Biology and National Clinical Research Center for Digestive Diseases, Xijing Hospital of Digestive Diseases, Fourth Military Medical University, Xi’an, China; ^2^School of Aerospace Medicine, Fourth Military Medical University, Xi’an, China; ^3^Department of Histology and Embryology, School of Basic Medicine, Xi’an Medical University, Xi’an, China; ^4^Treatment Centre for Traumatic Injures, Academy of Orthopedics Guangdong Province, The Third Affiliated Hospital of Southern Medical University, Guangzhou, China

**Keywords:** retroperitoneal liposarcomas, nomogram, prognostic factors, survival rate, SEER database

## Abstract

**Background:**

Retroperitoneal liposarcomas (RPLs), sarcoma of mesenchymal origin, are the most common soft tissue sarcomas (STS) of the retroperitoneum. Given the rarity of RPLs, the prognostic values of clinicopathological features in the patients remain unclear. The nomogram can provide a visual interface to aid in calculating the predicted probability that a patient will achieve a particular clinical endpoint and communication with patients.

**Methods:**

We included a total of 1,392 RPLs patients diagnosed between 2004 and 2015 from the Surveillance, Epidemiology, and End Results (SEER) database. For nomogram construction and validation, patients in the SEER database were divided randomly into the training cohort and internal validation cohort at a ratio of 7:3, while 65 patients with RPLs from our center between 2010 and 2016 served as the external validation cohort. The OS curves were drawn using the Kaplan–Meier method and assessed using the log-rank test. Moreover, Fine and Gray’s competing-risk regression models were conducted to assess CSS. Univariate and multivariate analyses were performed to select the prognostic factors for survival time. We constructed a predictive nomogram based on the results of the multivariate analyses.

**Results:**

Through univariate and multivariate analyses, it is found that age, histological grade, classification, SEER stage, surgery constitute significant risk factors for OS, and age, classification, SEER stage, AJCC M stage, surgery, and tumor size constitute risk factors for CSS. We found that the nomogram provided a good assessment of OS and CSS at 1, 3, and 5 years in patients with RPLs (1-year OS: (training cohort: AUC = 0.755 (*95% CI*, 0.714, 0.796); internal validation cohort: AUC = 0.754 (*95% CI*, 0.681, 0.827); external validation cohort: AUC = 0.793 (*95% CI*, 0.651, 0.935)); 3-year OS: (training cohort: AUC = 0.782 (*95% CI*, 0.752, 0.811); internal validation cohort: AUC = 0.788 (*95% CI*, 0.736, 0.841); external validation cohort: AUC = 0.863 (*95% CI*, 0.773, 0.954)); 5-year OS: (training cohort: AUC = 0.780 (*95% CI*, 0.752, 0.808); internal validation cohort: AUC = 0.783 (*95% CI*, 0.732, 0.834); external validation cohort: AUC = 0.854 (*95% CI*, 0.762, 0.945)); 1-year CSS: (training cohort: AUC = 0.769 (*95% CI*, 0.717, 0.821); internal validation cohort: AUC = 0.753 (*95% CI*, 0.668, 0.838); external validation cohort: AUC = 0.799 (*95% CI*, 0.616, 0.981)); 3-year CSS: (training cohort: AUC = 0.777 (*95% CI*, 0.742, 0.811); internal validation cohort: AUC = 0.787 (*95% CI*, 0.726, 0.849); external validation cohort: AUC = 0.808 (*95% CI*, 0.673, 0.943)); 5-year CSS: (training cohort: AUC = 0.773 (*95% CI*, 0.741, 0.805); internal validation cohort: AUC = 0.768 (*95% CI*, 0.709, 0.827); external validation cohort: AUC = 0.829 (*95% CI*, 0.712, 0.945))). The calibration plots for the training, internal validation, and external validation cohorts at 1-, 3-, and 5-year OS and CSS indicated that the predicted survival rates closely correspond to the actual survival rates.

**Conclusion:**

We constructed and externally validated an unprecedented nomogram prognostic model for patients with RPLs. The nomogram can be used as a potential, objective, and supplementary tool for clinicians to predict the prognosis of RPLs patients around the world.

## Introduction

Liposarcoma, beginning in the fat cells, is a relatively uncommon and heterogenous group of neoplasms, accounting for about 20% of all adult soft tissue sarcomas (STS) (1–3). Although the incidence of liposarcoma is low, overall liposarcomas affect a significant proportion of cancer patients. Ignored by the pharmaceutical industry as well as epidemiological, clinical, translational, and laboratory-based investigators, liposarcoma has a great influence on its overall outcome (4). Compared with well-studied cancers such as colorectal cancer, limited therapeutic options, high cost of treatment, and frequent misdiagnosis of liposarcoma cause a high burden on the health system (5–8). The retroperitoneum is the second most common location of liposarcoma after the extremities (9–11). Retroperitoneal liposarcomas (RPLs), with a higher recurrence rate and worse prognosis than extremity liposarcomas (ELs), are among the most challenging problems facing surgeons. RPLs respond poorly to most chemotherapeutic agents, and toxicity significantly limits the adequate dosing of radiation therapy (12–15). For most patients with RPLs, complete surgical resection represents the most effective treatment modality. Given the rarity of RPLs, the prognostic values of clinicopathological features in the patients remain unclear. Therefore, several studies have attempted to identify factors influencing RPL prognosis, including histologic subtypes, tumor grading, treatment strategy, tumor size, completeness of resection, and organ invasion (16–19). However, all these risk factors are difficult to answer the question asked by both clinicians and patients about survival rates, especially the survival time for each individual, and the limitations of these studies were very small sample sizes and lack of independent validation cohorts. As it happens, the nomogram, as a simple pictorial representation of a statistical prediction model to assist in clinical decision-making, generates a precise prediction based on the evaluation of important factors and provides accurate and individualized risk predictions for each individual for estimating the conditional risk of disease outcomes.

In this analysis, we aim to analyze and compare the prognostic features of RPLs using a relatively large number of cases obtained from the Surveillance, Epidemiology, and End Results (SEER) database and to develop a delicate nomogram to predict 1-, 3-, and 5-year overall survival (OS) and cancer-specific survival (CSS) based on significant prognostic factors. Further, we verified the prognostic value of the prediction model using an external validation set from our hospital database.

## Methods

### Data Source and Population Selection

This study used data from two sources. The first source was from the SEER database provided by the National Cancer Institute’s SEER*Stat software version 8.3.9.2 (https://seer.cancer.gov/data-software/). The screening of patients with RPLs was as follows: 1) patients came from the database of “SEER 18 Regs Custom Data (with additional treatment fields), Nov 2018 Sub (1975-2016 varying) database”; 2) the International Classification of Diseases for Oncology (ICD-O) site codes C48.0 (retroperitoneum) were used to identify patients; 3) according to “Histologic Type ICD-O-3,” the following pathological types were included in this study: liposarcoma (8850 to 8858); and 4) “Year of diagnosis” was set to 2004–2015. We only included patients positively diagnosed with histology tests. We excluded the patients with incomplete information, including demographic or survival information. Since the SEER database is publicly available and de-identified and the authors had no access to any participant-identifying information, it was not deemed necessary to obtain informed consent from the study population and local institutional review board review. The second source comprised of RPL patients who were diagnosed and received treatment at Xijing Hospital from 2010 to 2016. The included patients from our center were approved by the Institutional Review Board of Xijing Hospital, with orally informed consents. The study was performed in accordance with the ethical standards laid down in the 1964 declaration of Helsinki and its later amendments.

We extracted demographic information (age, sex, race), clinicopathological characteristics (histological grade (grade), morphology/pathological classification, tumor size, SEER stage, AJCC Stage, AJCC T stage, AJCC N stage, AJCC M stage), primary treatment modality (surgery and radiotherapy), survival time, vital status, and cause-specific death classification at the last follow-up from the chosen cases. The primary end point of this study was OS and CSS, and the effects were expressed as hazard ratios (*HRs*) with 95% confidence intervals (*CIs*). OS is defined as time to death from any cause, and CSS was defined as the time to death from RPLs. X-Tile software (version 3.6.1) was used to analyze the optimal cutoff point (65 years old) of age (20). Additionally, RPL histology was categorized as well-differentiated (WDLS), myxoid (MLS), pleomorphic (PLS), dedifferentiated (DDLS), and other (round cell, mixed, angiomyoliposarcoma, fibroblastic, and not otherwise specified) liposarcomas according to WHO classification (1). Race was categorized as white, black, or other (American Indian/Alaska Native, Asian or Pacific Islander, Hispanic).

### Statistical Analysis

Statistical analyses were performed using SPSS 20.0 (SPSS, Inc., Chicago, IL) and R software (version 4.1.2). Descriptive statistics are presented as proportions (*%*) and frequencies (*n*). To estimate cancer survival probabilities, we considered cancer death as the event of interest and non-cancer death as the censored observation. We used Fine and Gray’s competing risk analysis (21) to estimate the cumulative incidence function (CIF) to explore each single-variable incidence of each competing event. Moreover, we used the proportional sub-distribution hazard model to identify the significant variables associated with CSS and the competing risk nomogram was constructed based on these factors to assess the association between predictor variables and the outcomes. The OS curves were drawn using the Kaplan–Meier method and assessed using the log-rank test. The significant prognostic variables (*P* < 0.05) were selected by the univariate Cox proportional hazard model and further by the multivariate Cox proportional hazard model to build their covariate-adjusted effects on survival time. Multivariate analyses were performed with the backward stepwise regression to identify independent risk factors, and the nomogram for OS was constructed based on these factors. Variables selected for inclusion were carefully chosen to ensure parsimony of the final models. The proportional hazard assumptions were checked using examining scaled Schoenfeld residuals (22) and violation of the proportional hazard assumptions was not observed.

For nomogram construction and validation, patients in the SEER database were divided randomly into the training cohort and internal validation cohort at a ratio of 7:3, while those in our hospital patient data set served as the external validation cohort. All incorporating prognostic variables from the training cohort were included to build the nomogram for predicting the probability of a patient’s survival rate at 1, 3, or 5 years. Each subtype of the factors on the nomogram corresponds to a point on the “Point” scale. The points for each variable are summed together to generate a total-point score. The total-point scores projected on the bottom scales indicate the probabilities of 1-, 3-, and 5-year OS or CSS. Validation of each nomogram included three procedures in the training, internal validation, and external validation cohorts. First, the discrimination performance of the nomogram was evaluated using the area under the curve (AUC) value of the receiver operating characteristic (ROC), which ranges from 0.5 (chance discrimination) to 1.0 (perfect discrimination, equivalent to the standard). Second, the calibration plot was conducted using a bootstrap method with 1,000 resamples to compare the consistency between actual observed survival rates and predicted survival rates. Intuitively, the closer the simple regression line between the actual and predicted survival rates is to the diagonal line, the closer the predicted survival rates to the actual survival rates. All statistical tests used a significance level of 5% in a two-tailed test.

## Results

### Clinical Characteristics

After excluding patients with missing follow-up data, a total of 1,392 RPLs patients diagnosed between 2004 and 2015 from the SEER database were selected and assigned to the training cohort (n = 974) and the internal validation cohort (n = 418). Based on the same inclusion and exclusion criteria as used in the SEER cohort, 65 patients with RPLs were included in the external validation cohort from the Xijing Hospital. Of the total SEER group, the demographic and clinical characteristics did not differ between the training and internal validation cohorts. The general demographic and clinicopathological features of patients from the SEER database are summarized in **Table 1**. Patients diagnosed with WDLS (*n* = 437), MLS (*n* = 86), PLS (*n* = 39), DDLS (*n* = 523), and the category of other liposarcomas (*n* = 307) were included in the study for comparisons. Most patients with RPLs (84.19%) underwent surgery, of which 501 (35.99%) were subjected to partial excision, 182 (13.07%) to total excision, and 489 (35.13%) to radical surgery. Nearly half of the operations addressed complete excision of the lesion. A tumor size larger than 10 cm comprises the majority of RPLs. **Supplementary Table 1** displays the general demographic and clinicopathological features of patients chosen from Xijing Hospital. Patients diagnosed with WDLS (*n* = 22), MLS (*n* = 8), PLS (*n* = 9), DDLS (*n* = 23), and the category of other liposarcomas (*n* = 3) were included in the study for comparisons. Among them, The WDLS/DDLS subtypes were more prevalent histologic subtypes (33.85% and 35.38%, respectively). Concerning the treatment strategy, most patients with RPLs (90.77%) underwent surgery, and only 6 (9.23%) patients with RPLs did not have surgery. This result is consistent with the SEER database.

**Table 1:** Demographics and clinicopathologic characteristics of SEER Patients with RPLs.

Category	Training cohort (*n* = 974)			Internal validation cohort (*n* = 418)			Total cohort (*n* = 1,392)		*P*
	n	%		n	%		n	%	
**Age**							
<65	490	50.31%		218	52.15%		708	50.86%	0.559
≥65	484	49.69%		200	47.85%		684	49.14%
**Sex**							
Female	441	45.28%		169	40.43%		610	43.82%	0.099
Male	533	54.72%		249	59.57%		782	56.18%
**Race**							
White	811	83.26%		362	86.60%		1,173	84.27%	0.141
Black	65	6.67%		17	4.07%		82	5.89%
Other*^a^*	98	10.06%		39	9.33%		137	9.84%
**Histological grade***^b^*							
I/II	499	51.23%		204	48.80%		703	50.50%	0.706
III/IV	336	34.50%		152	36.36%		488	35.06%
Unknown	139	14.27%		62	14.83%		201	14.44%
**Classification**							
WDLS	302	31.01%		135	32.30%		437	31.39%	0.624
MLS	66	6.78%		20	4.78%		86	6.18%
PLS	29	2.98%		10	2.39%		39	2.80%
DDLS	366	37.58%		157	37.56%		523	37.57%
Other*^c^*	211	21.66%		96	22.97%		307	22.05%
**Seer stage**							
Localized	436	44.76%		200	47.85%		636	45.69%	0.370
Regional	388	39.84%		146	34.93%		534	38.36%
Distant	107	10.99%		50	11.96%		157	11.28%
Unknown	43	4.41%		22	5.26%		65	4.67%
**AJCC stage**							
I/II	443	45.48%		178	42.58%		621	44.61%	0.195
III/IV	361	37.06%		150	35.89%		511	36.71%
Unknown	170	17.45%		90	21.53%		260	18.68%
**AJCC T stage**							
T1	39	4.00%		24	5.74%		63	4.53%	0.255
T2	838	86.04%		347	83.01%		1,185	85.13%
Unknown	97	9.96%		47	11.24%		144	10.34%
**AJCC N stage**							
N0	889	91.27%		374	89.47%		1,263	90.73%	0.565
N1	24	2.46%		12	2.87%		36	2.59%
Unknown	61	6.26%		32	7.66%		93	6.68%
**AJCC M stage**							
M0	876	89.94%		371	88.76%		1,247	89.58%	0.158
M1	65	6.67%		24	5.74%		89	6.39%
Unknown	33	3.39%		23	5.50%		56	4.02%
**Surgery**							
No surgery	122	12.53%		61	14.59%		183	13.15%	0.519
Partial excision	342	35.11%		159	38.04%		501	35.99%
Total excision	133	13.66%		49	11.72%		182	13.07%
Radical surgery	350	35.93%		139	33.25%		489	35.13%
Unknown	27	2.77%		10	2.39%		37	2.66%
**Radiation recode**							
No	765	78.54%		331	79.19%		1,096	78.74%	0.830
Yes	209	21.46%		87	20.81%		296	21.26%
**Tumor size**							
<10 cm	134	13.76%		66	15.79%		200	14.37%	0.560
10–20 cm	351	36.04%		136	32.54%		487	34.99%	
>20 cm	418	42.92%		183	43.78%		601	43.18%	
Unknown	71	7.29%		33	7.89%		104	7.47%	

^a^American Indian/AK Native, Asian/Pacific Islander, unknown.

^b^Grade I: well differentiated; grade II: moderately differentiated; grade III: poorly differentiated; grade IV: undifferentiated.

^c^Round cell liposarcoma, mixed liposarcoma, angiomyoliposarcoma, fibroblastic liposarcoma, and not otherwise specified liposarcoma.

the SEER database, the Surveillance, Epidemiology, and End Results database; WDLS, well-differentiated liposarcoma; MLS, myxoid liposarcoma; PLS, pleomorphic liposarcoma; DDLS, dedifferentiated liposarcoma; cm, centimeter.

### Survival Analysis

The Kaplan–Meier survival curves of OS for patients by age, sex, race, grade, classification, SEER stage, AJCC stage, AJCC T stage, AJCC N stage, AJCC M stage, surgical options, radiation recode, and tumor size are depicted in **Supplementary Figure 1**. Kaplan–Meier survival curves indicated that patients with older age, male, higher grade, higher AJCC stage, AJCC N1 stage, AJCC M1 stage, and increased severity of the SEER stage had a relatively poor OS, while patients who underwent surgery had a beneficial effect on OS compared with no surgery. As for histologic classification, patients with WDLS had significantly longer OS compared to patients with the MLS (*P* = 0.001), PLS (*P* < 0.001), DDLS (*P* < 0.001), and other liposarcomas (*P* < 0.001).

The cumulative incidence function curves of CSS for patients by age, sex, race, grade, classification, SEER stage, AJCC stage, AJCC T stage, AJCC N stage, AJCC M stage, surgical options, radiation recode, and tumor size are depicted in **Supplementary Figure 2**. Variables regarding older age, male, higher grade, higher AJCC stage, AJCC N1 stage, AJCC M1 stage, enlarged tumor, and increased severity of the SEER stage had a relatively poor CSS, while patients who underwent surgery had a beneficial effect on CSS compared with no surgery. As for histologic classification, patients with WDLS had significantly longer CSS compared to patients with MLS (*P* < 0.001), PLS (*P* < 0.001), DDLS (*P* < 0.001), and other liposarcomas (*P* < 0.001).

Univariate analyses of variables potentially influencing OS and CSS are summarized in **Table 2**. Factors including age, sex, grade, classification, SEER stage, AJCC stage, AJCC N stage, AJCC M stage, surgery, and radiation recode were significantly related to OS through univariate Cox proportional hazard regression analysis, while factors including age, sex, grade, classification, SEER stage, AJCC stage, AJCC N stage, AJCC M stage, surgery, and tumor size were significantly associated with CSS through univariate competing analysis. The variables that were identified significant with univariate analysis were used for subsequent multivariate analysis (**Table 3**). After adjustment for possible confounders, we considered that age, grade, classification, SEER stage, and surgery constitute significant risk factors for OS and age, classification, SEER stage, AJCC M stage, surgery, and tumor size constitute significant risk factors for CSS in the multivariable analysis.

**Table 2:** Univariate analysis for overall survival and cancer-specific survival in the training cohort.

Category	*n*	Overall survival *P* (log-rank test)	Cancer-specific survival *P* (Fine and Gray’s test)
**Age**			
<65	491	Ref.	Ref.
≥65	483	<0.001	0.004
**Sex**			
Female	441	Ref.	Ref.
Male	533	<0.001	0.022
**Race**			
White	811	Ref.	Ref.
Black	65	0.548	0.553
Other*^a^*	98	0.440	0.989
**Histological grade***^b^*			
I/II	499	Ref.	Ref.
III/IV	336	<0.001	<0.001
**Classification**			
WDLS	302	Ref.	Ref.
MLS	66	0.007	<0.001
PLS	29	<0.001	<0.001
DDLS	366	<0.001	<0.001
Other*^c^*	211	<0.001	<0.001
**Seer stage**			
Localized	436	Ref.	Ref.
Regional	388	<0.001	<0.001
Distant	107	<0.001	<0.001
**AJCC stage**			
I/II	443	Ref.	Ref.
III/IV	361	<0.001	<0.001
**AJCC T stage**			
T1	39	Ref.	Ref.
T2	838	0.926	0.196
**AJCC N stage**			
N0	889	Ref.	Ref.
N1	24	<0.001	<0.001
**AJCC M stage**			
M0	876	Ref.	Ref.
M1	65	<0.001	<0.001
**Surgery**			
No surgery	122	Ref.	Ref.
Partial excision	342	<0.001	<0.001
Total excision	133	<0.001	<0.001
Radical surgery	350	<0.001	<0.001
**Radiation recode**			
No	765	Ref.	Ref.
Yes	209	0.026	0.315
**Tumor size**			
<10 cm	134	Ref.	Ref.
10–20 cm	351	0.875	0.053
>20 cm	418	0.487	0.001

^a^American Indian/AK Native, Asian/Pacific Islander, unknown.

^b^Grade I: well differentiated; grade II: moderately differentiated; grade III: poorly differentiated; grade IV: undifferentiated.

^c^Round cell liposarcoma, mixed liposarcoma, angiomyoliposarcoma, fibroblastic liposarcoma and not otherwise specified liposarcoma.

The P value in the column of univariate analysis means that the variable was selected in the next multivariate analysis.

RPLs, retroperitoneal liposarcomas; WDLS, well-differentiated liposarcoma; MLS, myxoid liposarcoma; PLS, pleomorphic liposarcoma; DDLS, dedifferentiated liposarcoma; Ref., referent.

**Table 3:** Multivariate analysis for overall survival and cancer-specific survival in the training cohort.

Category	Overall survival		Cancer-specific survival	
	*HR (95% CI)*	*P*	*HR (95% CI)*	*P*
**Age**				
<65	Ref.	—	Ref.	—
≥65	1.59 (1.31, 1.93)	<0.001	1.43 (1.11, 1.83)	0.005
**Sex**				
Female	Ref.	—	Ref.	—
Male	1.16 (0.96, 1.41)	0.128	0.96 (0.74, 1.24)	0.740
**Histological grade***^a^*				
I/II	Ref.	—	Ref.	—
III/IV	1.87 (1.11, 3.15)	0.018	1.55 (0.80, 2.98)	0.190
**Classification**				
WDLS	Ref.	—	Ref.	—
MLS	1.63 (1.10, 2.41)	0.015	2.26 (1.27, 4.04)	0.005
PLS	1.31 (0.75, 2.30)	0.344	1.48 (0.65, 3.36)	0.350
DDLS	1.75 (1.31, 2.32)	<0.001	2.34 (1.47, 3.72)	<0.001
Other*^b^*	1.47 (1.14, 1.90)	0.003	1.63 (1.04, 2.55)	0.032
**Seer stage**				
Localized	Ref.	—	Ref.	—
Regional	1.33 (1.07, 1.64)	0.009	1.35 (1.02, 1.79)	0.038
Distant	1.75 (1.1, 2.78)	0.018	2.28 (1.32, 3.94)	0.003
**AJCC stage**				
I/II	Ref.	—	Ref.	—
III/IV	1.07 (0.65, 1.77)	0.791	1.68 (0.93, 3.03)	0.084
**AJCC T stage**				
T1	—	—	Ref.	—
T2	—	—	0.89 (0.48, 1.65)	0.710
**AJCC N stage**				
N0	Ref.	—	Ref.	—
N1	1.20 (0.69, 2.09)	0.530	1.58 (0.88, 2.84)	0.130
**AJCC M stage**				
M0	Ref.	—	Ref.	—
M1	1.40 (0.77, 2.55)	0.274	1.96 (1.18, 3.25)	0.009
**Surgery**				
No surgery	Ref.	—	Ref.	—
Partial excision	0.29 (0.20, 0.42)	<0.001	0.21 (0.13, 0.33)	<0.001
Total excision	0.34 (0.23, 0.51)	<0.001	0.28 (0.16, 0.46)	<0.001
Radical surgery	0.29 (0.20, 0.42)	<0.001	0.22 (0.14, 0.36)	<0.001
**Radiation recode**				
No	Ref.	—	—	—
Yes	0.86 (0.68, 1.09)	0.226	—	—
**Tumor size**				
<10 cm	—	—	Ref.	—
10–20 cm	—	—	1.24 (0.78, 1.96)	0.368
>20 cm	—	—	1.91 (1.22, 2.98)	0.005

^a^Grade I: well differentiated; grade II: moderately differentiated; grade III: poorly differentiated; grade IV: undifferentiated.

^b^Round cell liposarcoma, mixed liposarcoma, angiomyoliposarcoma, fibroblastic liposarcoma, and not otherwise specified liposarcoma.

RPLs, retroperitoneal liposarcomas; WDLS, well-differentiated liposarcoma; MLS, myxoid liposarcoma; PLS, pleomorphic liposarcoma; DDLS, dedifferentiated liposarcoma; HR, hazard ratio; CI, confidence interval; Ref., referent.

### Nomogram Construction and Validation

**Figure 1:**
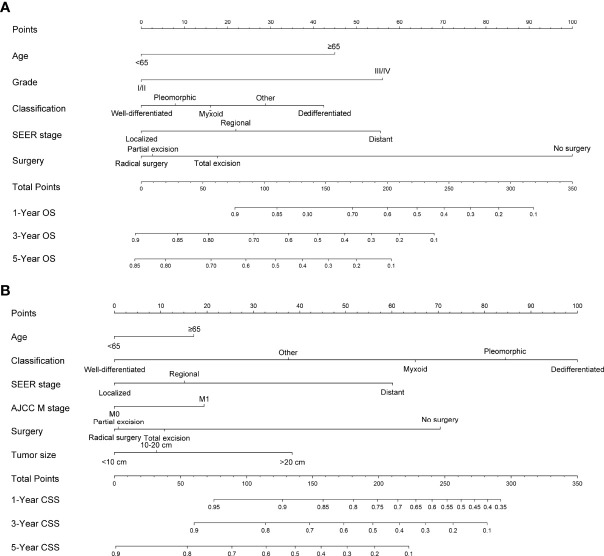
Nomogram predicting 1-, 3-, and 5-year **(A)** OS, **(B)** and CSS of patients with RPLs. Summarizing the scores of each variable together and the total points projected on the bottom scales indicate the probabilities of 1-, 3-, and 5-year OS and CSS. OS, overall survival; CSS, cancer-specific survival; RPLs, retroperitoneal liposarcomas.

**Figure 2:**
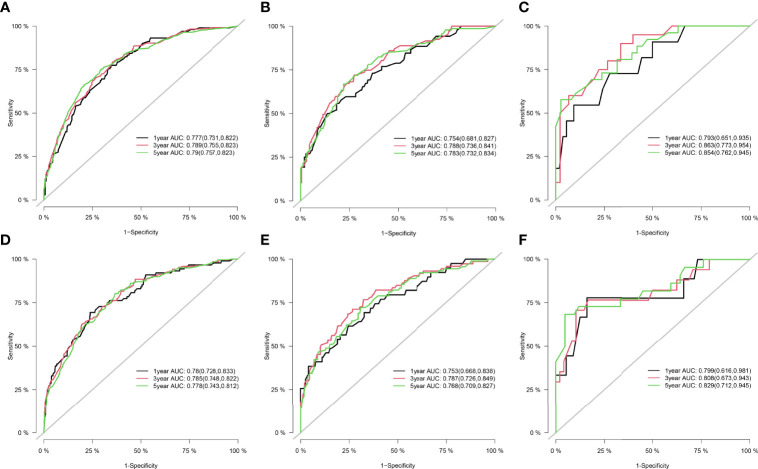
ROC curve for the nomograms in predicting prognosis in patients with RPLs. **(A)** ROC of 1-, 3-, and 5-year OS in the training cohort. **(B)** ROC of 1-, 3-, and 5-year OS in the internal validation cohort. **(C)** ROC of 1-, 3-, and 5-year OS in the external validation cohort. **(D)** ROC of 1-, 3-, and 5-year CSS in the training cohort. **(E)** ROC of 1-, 3-, and 5-year CSS in the internal validation cohort. **(F)** ROC of 1-, 3-, and 5-year CSS in the external validation cohort. ROC, receiver operating characteristic; OS, overall survival; CSS, cancer-specific survival; RPLs, retroperitoneal liposarcomas.

**Figure 3:**
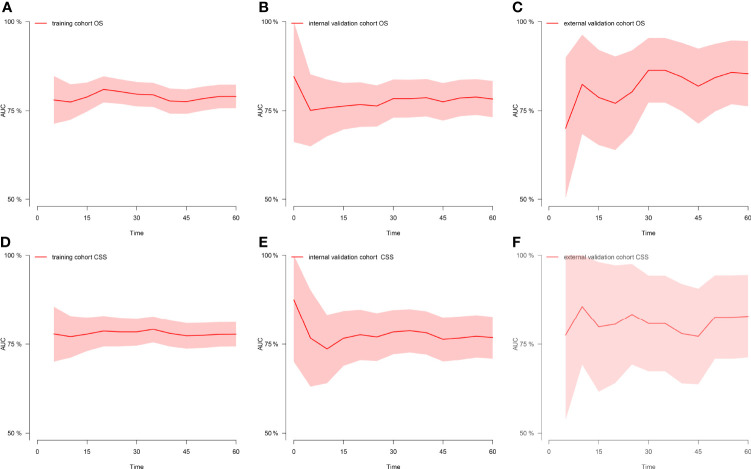
Time-dependent AUC at each time point. **(A)** Time-dependent AUC of OS in the training cohort. **(B)** Time-dependent AUC of OS in the internal validation cohort. **(C)** Time-dependent AUC of OS in the external validation cohort. **(D)** Time-dependent AUC of CSS in the training cohort. **(E)** Time-dependent AUC of CSS in the internal validation cohort. **(F)** Time-dependent AUC of CSS in the external validation cohort. AUC, the area under the curve value of the receiver operating characteristic; OS, overall survival; CSS, cancer-specific survival.

**Figure 4:**
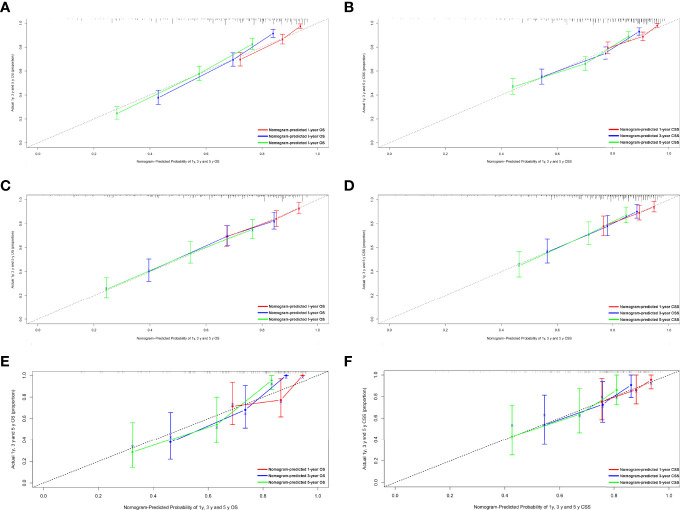
Calibration plots of the nomogram for 1-, 3-, and 5-year OS and CSS prediction. **(A)** Calibration curves of 1-, 3-, and 5-year OS in the training cohort. **(B)** Calibration curves of 1-, 3-, and 5-year CSS in the training cohort. **(C)** Calibration curves of 1-, 3-, and 5-year OS in the internal validation cohort. **(D)** Calibration curves of 1-, 3-, and 5-year CSS in the internal validation cohort. **(E)** Calibration curves of 1-, 3-, and 5-year OS in the external validation cohort. **(F)** Calibration curves of 1-, 3-, and 5-year CSS in the external validation cohort. OS, overall survival; CSS, cancer-specific survival.

**Figure 1** shows the nomogram of the prognosis of 1-, 3-, and 5-year OS and CSS. Our nomogram showed good discrimination and prediction capabilities. The predictive performance of the nomogram for 1-, 3-, and 5- year OS and CSS in the training, internal validation, and external validation cohorts was evaluated by the ROC curve. We found that the nomogram provided a good assessment of OS and CSS at 1, 3, and 5 years in patients with RPLs [1-year OS: (training cohort: AUC = 0.755 (*95% CI*, 0.714, 0.796); internal validation cohort: AUC = 0.754 (*95% CI*, 0.681, 0.827); external validation cohort: AUC = 0.793 (*95% CI*, 0.651, 0.935)]; 3-year OS: [training cohort: AUC = 0.782 (*95% CI*, 0.752, 0.811); internal validation cohort: AUC = 0.788 (*95% CI*, 0.736, 0.841); external validation cohort: AUC = 0.863 (*95% CI*, 0.773, 0.954)]; 5-year OS: [training cohort: AUC = 0.780 (*95% CI*, 0.752, 0.808); internal validation cohort: AUC = 0.783 (*95% CI*, 0.732, 0.834); external validation cohort: AUC = 0.854 (*95% CI*, 0.762, 0.945)]; 1-year CSS: [training cohort: AUC = 0.769 (*95% CI*, 0.717, 0.821); internal validation cohort: AUC = 0.753 (*95% CI*, 0.668, 0.838); external validation cohort: AUC = 0.799 (*95% CI*, 0.616, 0.981)]; 3-year CSS: [training cohort: AUC = 0.777 (*95% CI*, 0.742, 0.811); internal validation cohort: AUC = 0.787 (*95% CI*, 0.726, 0.849); external validation cohort: AUC = 0.808 (*95% CI*, 0.673, 0.943)]; 5-year CSS: [training cohort: AUC = 0.773 (*95% CI*, 0.741, 0.805); internal validation cohort: AUC = 0.768 (*95% CI*, 0.709, 0.827); external validation cohort: AUC = 0.829 (*95% CI*, 0.712, 0.945)]. The results are illustrated in **Figure 2** and **Table 4**. **Figure 3** shows the time-dependent AUC at each time point. The results revealed that the model had a higher AUC at all time points. **Figure 4** shows the calibration plots for the training, internal validation, and external validation cohorts at 1-, 3-, and 5- year OS and CSS. The results indicated that the predicted survival rates of 1, 3, and 5 years closely correspond to the actual survival rates.

**Table 4:** AUC for the nomogram in patients with RPLs.

Survival		Training cohort	Internal validation cohort	External validation cohort
Overall survival	At 1 year	0.755 (*95% CI*, 0.714, 0.796)	0.754 (*95% CI*, 0.681, 0.827)	0.793 (*95% CI*, 0.651, 0.935)
	At 3 years	0.782 (*95% CI*, 0.752, 0.811)	0.788 (*95% CI*, 0.736, 0.841)	0.863 (*95% CI*, 0.773, 0.954)
	At 5 years	0.780 (*95% CI*, 0.752, 0.808)	0.783 (*95% CI*, 0.732, 0.834)	0.854 (*95% CI*, 0.762, 0.945)
Cancer-specific survival	At 1 year	0.769 (*95% CI*, 0.717, 0.821)	0.753 (*95% CI*, 0.668, 0.838)	0.799 (*95% CI*, 0.616, 0.981)
	At 3 years	0.777 (*95% CI*, 0.742, 0.811)	0.787 (*95% CI*, 0.726, 0.849)	0.808 (*95% CI*, 0.673, 0.943)
	At 5 years	0.773 (*95% CI*, 0.741, 0.805)	0.768 (*95% CI*, 0.709, 0.827)	0.829 (*95% CI*, 0.712, 0.945)

RPLs, retroperitoneal liposarcomas; CI, confidence interval; AUC, the area under the curve value of the receiver operating characteristic.

## Discussion

RPLs, sarcoma of mesenchymal origin, are the most common STS of the retroperitoneum (23–25). RPLs account for approximately 40% of cases of STS in the retroperitoneum (26, 27). Several studies have suggested that the surgical management and histologic subtype of RPLs are associated with prognosis in patients with RPLs (28–32). Given the rarity of RPLs, the prognostic values of clinicopathological features in the patients remain unclear. Meanwhile, RPLs continue to pose a challenge with prediction of clinical behavior. Previous studies have evaluated prognostic factors affecting prognosis in the patients with RPLs, but the sample size of these studies was very small, and none have included more than a few hundred patients (32–35). Although these findings advance our understanding of RPLs, they may be especially vulnerable to institutional bias, and they still require additional information on this uncommon malignancy. The strengths of the present population-based study of prognosis in the patients with RPLs include its large size and generalizability beyond a few institutions. With 1,392 patients with RPLs at baseline with follow-up data, we identified the prognostic factors of patients in RPLs. OS at 5 years was 51% and CSS at 5 years was 63% in the study, which is consistent with the results of other recent studies (25, 28, 36, 37). Our work analyzes and compares the prognostic features of RPLs using a relatively large number of cases and develops a delicate nomogram to predict 1-, 3-, and 5-year OS and CSS based on significant prognostic factors.

In this study, we retrospectively assessed the clinicopathological characteristics of patients with RPLs and identified the related risk factors for patients’ prognosis. Our analyses identified histologic grade, the indicator of tumor aggressiveness, as predictors of survival in patients with RPLs. The finding is consistent with previous research showing that increased survival is directly associated with better histologic grade (35, 37–40). Furthermore, previous analyses showed that completeness of resection is another important prognostic factor (17, 40–43). We also found that the prognosis of patients who underwent resection was significantly better than that of patients without surgical resection, especially that of patients with radical surgery. Additionally, we found that patients with a distant and regional SEER stage had poor OS and CSS than those with a localized SEER stage. As the other authors suggested, patients with tumors invading adjacent structures may be more likely to develop residual microscopic or gross disease after resection (17, 31, 41, 44). In this study, histological subtype was also an important indicator of prognosis. The WDLS/DDLS subtypes were more prevalent histologic subtypes in the present series. Patients with the WDLS subtype had the best prognosis, while patients with the DDLS subtype had the worst prognosis (32, 45). DDLS may be difficult to identify at the time of presentation because they exhibit a variable histologic picture (46, 47). Thus, careful and extensive sampling is mandatory in each patient. Other subtypes, such as MLS and PLS, were rare as reported previously (46, 48). Also interesting is the finding that tumor size is an independent risk factor for CSS, not for OS. The 5-cm threshold used in the AJCC staging system is still limited about the value for RPLs because such small RPLs are uncommon in the present and other international studies (17, 25, 35, 43). Meanwhile, several studies proposed that the optimal threshold in tumor sizes should be revised upward to 10 cm (25, 36). In this study, we rigorously evaluated the relationships between tumor size and prognosis in a large cohort of patients with RPLs, using several different cut points for dichotomization. In all of these analyses, tumor size was not associated with OS. However, when the cut points were set at 10 and 20 cm, larger tumor size was associated with poor CSS. Therefore, the role of the AJCC T-classification system in predicting the prognosis of RPL patients should be interpreted with caution.

Although the multivariate analysis confirmed the related risk factors for patients’ prognosis, these variables have not been able to produce an accurate and discriminatory prediction for RPLs, especially estimating the survival rates of each individual. Thus, a specially designed prognostic prediction model is needed to answer this question. The nomogram generates a precise prediction based on the evaluation of important factors and provide accurate and individualized risk predictions for each individual for estimating the conditional risk of disease outcomes. Although an article reported that the nomogram can accurately estimate the recurrence-free survival (RFS) of patients with RPLs (35), the sample size was relatively small and patients were taken from a single institution. Studies including a bigger sample size from multicenters were necessary. Furthermore, there are no studies that construct a nomogram that can estimate the OS and CSS of patients with RPLs. Our study fills this gap at least partially by creating nomogram models to establish the OS and CSS of RPLs based on a large database. We constructed and validated a nomogram for predicting the 1-, 3-, and 5-year OS and CSS in patients with RPLs. For nomogram construction and validation, patients in the SEER database were divided randomly into the training cohort and internal validation cohort at a ratio of 7:3, while those in our hospital patient data set served as the external validation cohort. Through univariate and multivariate analyses, it is found that age, grade, classification, SEER stage, and surgery constitute significant risk factors for OS, and age, classification, SEER stage, AJCC M stage, surgery, and tumor size constitute risk factors for CSS. We found that the nomogram provided a good assessment of OS and CSS at 1, 3, and 5 years in patients with RPLs and the model had a higher AUC at all time points. In addition, the calibration plots indicated that the predicted survival rates of 1, 3, and 5 years closely correspond to the actual survival rates. Therefore, the nomogram can be used to better assess an individual clinical outcome. Meanwhile, the nomogram can be easily applied to abundant settings, such as clinic, bedside, or at home, without depending on the computer. With simple training, healthcare professionals, patients, and the public can quickly grasp the nomogram to assess the individualized risk predictions for each individual. Using the nomogram, it can be seen that an individual aged 70 (45 points for OS; 18 points for CSS), diagnosed with DDLS (42 points for OS; 100 points for CSS), grade III (56 points for OS), localized SEER stage (0 points for OS; 0 points for CSS), and AJCC M1 stage (20 points for CSS) and who has been treated with total excision (18 points for OS; 10 points for CSS), has a total point score of 161 for OS and 148 for CSS. This equates to 1-, 3-, and 5-year OS of 0.73, 0.41, and 0.27, and 1-, 3-, and 5-year CSS of 0.86, 0.69, and 0.42, respectively.

There are several limitations that should be considered when interpreting our results. Firstly, it is difficult to avoid selection bias because the study was a retrospective study using public databases. Second, our nomogram provided individual predictions of OS for patients with five clinicopathological factors, and CSS with six clinicopathological factors, lacking other additional variables such as PD-1, vimentin, and Ki-67. The levels of PD-1, vimentin, and Ki-67 expression have been found to increase in patients with RPLs and were associated with poor CSS and RFS (27b; 31, 49, 50). However, the SEER database lacks these variables and future studies are warranted to further incorporate these variables into analysis. Third, when we included treatment as a prognostic factor, we only considered the effects of surgery and radiotherapy on prognosis, neglecting adjuvant chemotherapy and other medical therapies, such as target therapies, even though most of the research indicates that adjuvant chemotherapy has little to offer for patients with RPLs (17, 48, 51, 52). Fourth, the SEER provided no information regarding the margin of resection for patients with RPLs. The margin of resection might be presented with a prognostic value in RPL patients. Thus, we will focus on it in the future research. Fifth, the sample size of the validation cohort was small and patients were taken from a single center. Although the verification results were good, the results of AUC might change after increasing the sample size and the number of centers. Future studies need to include validation cohorts with a larger sample size from multicenters. Fifth, DDLS may be difficult to identify at the time of presentation, especially in those institutions with lack of specialist expertise in treating RPLs. However, misclassifications would affect study results and tend to obscure differences rather than exaggerate them (25). Therefore, further studies are needed to examine the effects of these factors on the prognosis to provide guidelines for the treatment of RPLs.

## Conclusion

In conclusion, age, grade, classification, SEER stage, and surgery constitute significant risk factors for OS of patients with RPLs, and age, classification, SEER stage, AJCC M stage, surgery, and tumor size constitute risk factors for CSS. We constructed and validated a nomogram for predicting the OS and CSS in patients with RPLs. This nomogram that provided individual predictions of OS for patients with five clinicopathological factors and CSS with six clinicopathological factors can be used as a potential, objective, and supplementary tool for clinicians to predict the prognosis of RPL patients around the world.

## Data Availability Statement

The raw data supporting the conclusions of this article will be made available by the authors, without undue reservation.

## Ethics Statement

The studies involving human participants were reviewed and approved by the Institutional Review Board of Xijing Hospital. Written informed consent for participation was not required for this study in accordance with the national legislation and the institutional requirements.

## Author Contributions

YL and GW participated in the design of this study and wrote the manuscript. LH and DF conceived the original idea and supervised the overall direction and planning of the project. YZ, WY, XW, LD, LN, JC, WZ, JL, and HZ contributed to the acquisition of the data, analysis, and interpretation of the data. All authors contributed to the article and approved the submitted version.

## Funding

This project was supported by a project supported by the National Natural Science Foundation of China (No. 82073210), the grant of Shaanxi Province (No. 2019ZDLSF01-02-01), and the Xjijng Zhutui Project. The funding bodies played no role in the design of the study and collection, analysis, and interpretation of data and in writing the manuscript.

## Conflict of Interest

The authors declare that the research was conducted in the absence of any commercial or financial relationships that could be construed as a potential conflict of interest.

## Publisher’s Note

All claims expressed in this article are solely those of the authors and do not necessarily represent those of their affiliated organizations, or those of the publisher, the editors and the reviewers. Any product that may be evaluated in this article, or claim that may be made by its manufacturer, is not guaranteed or endorsed by the publisher.
