# Supplementary material for: Development and Validation of a Prognostic Model to Predict the Prognosis of Patients With Retroperitoneal Liposarcoma: A Large International Population-Based Cohort Study
Source: Front Oncol. 2022 Jun 2;12:857827. doi: 10.3389/fonc.2022.857827 (PMC9201285; doi:10.3389/fonc.2022.857827)
Supplement: Supplementary file 2 [file DataSheet_2.docx]

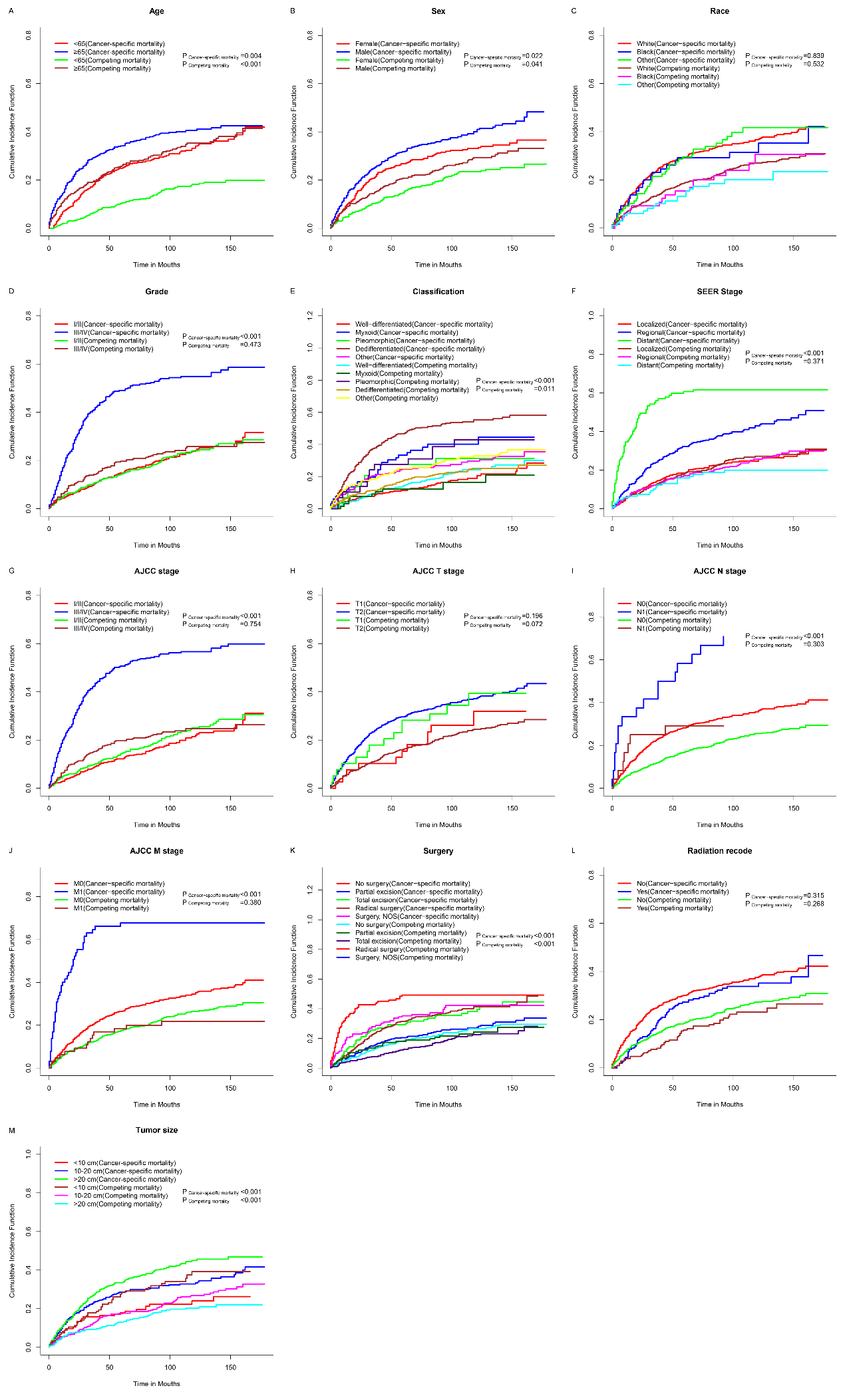


**Supplementary Figure 2** Cumulative incidence function curves of CSS according to (A) Age, (B) Sex, (C) Race, (D) Grade, (E) Classification, (F) SEER stage, (G) AJCC stage, (H) AJCC T stage, (I) AJCC N stage, (G) AJCC M stage, (K) Surgery, (L) Radiation recode, (M) Tumor size. Abbreviations: CSS: cancer-specific survival.
